# Single nucleotide polymorphism mining and nucleotide sequence analysis of *Mx1* gene in exonic regions of Japanese quail

**DOI:** 10.14202/vetworld.2015.1435-1443

**Published:** 2015-12-29

**Authors:** Diwesh Kumar Niraj, Pushpendra Kumar, Chinmoy Mishra, Raj Narayan, Tarun Kumar Bhattacharya, Kush Shrivastava, Bharat Bhushan, Ashok Kumar Tiwari, Vishesh Saxena, Nihar Ranjan Sahoo, Deepak Sharma

**Affiliations:** 1 Animal Genetics Division, Indian Veterinary Research Institute, Izatnagar, Bareilly - 243 122, Uttar Pradesh, India; 2 Department of Animal Genetics and Breeding, College of Veterinary Science and Animal Husbandry, Orissa University of Agriculture and Technology, Bhubaneswar, Odisha, India; 3 Department of Avian Genetics and Breeding, Central Avian Research Institute, Izatnagar, Bareilly, 243 122, Uttar Pradesh, India; 4 Directorate of Poultry Research, Rajendranagar, Hyderabad, Telangana, India; 5 Standardization Division, Indian Veterinary Research Institute, Izatnagar, Bareilly - 243 122, Uttar Pradesh, India

**Keywords:** Japanese quail, *Mx1* gene, nucleotide sequencing, polymorphism, polymerase chain reaction-single-strand conformation polymorphism

## Abstract

**Aim:**

An attempt has been made to study the Myxovirus resistant (*Mx1*) gene polymorphism in Japanese quail.

**Materials and Methods:**

In the present, investigation four fragments *viz*. Fragment I of 185 bp (Exon 3 region), Fragment II of 148 bp (Exon 5 region), Fragment III of 161 bp (Exon 7 region), and Fragment IV of 176 bp (Exon 13 region) of *Mx1* gene were amplified and screened for polymorphism by polymerase chain reaction-single-strand conformation polymorphism technique in 170 Japanese quail birds.

**Results:**

Out of the four fragments, one fragment (Fragment II) was found to be polymorphic. Remaining three fragments (Fragment I, III, and IV) were found to be monomorphic which was confirmed by custom sequencing. Overall nucleotide sequence analysis of *Mx1* gene of Japanese quail showed 100% homology with common quail and more than 80% homology with reported sequence of chicken breeds.

**Conclusion:**

The *Mx1* gene is mostly conserved in Japanese quail. There is an urgent need of comprehensive analysis of other regions of *Mx1* gene along with its possible association with the traits of economic importance in Japanese quail.

## Introduction

The brisk increase in human population during the last few decades led to hassled research on improving production performance of livestock and poultry to meet the requirement of quality food [1]. However, increase in production performance is mostly coupled with compromised health-related traits due to their negative genetic correlation [2,3]. The poultry industry has been facing intimidating losses due to rise in the incidence of diseases associated with the intensive management system. Conventional vaccinations coupled with the modern managemental practices strive to protect the birds from many pathogens due to change in pathogenicity of causative agents, emerging of resistant strains, and sometime ineffective medical treatments. Hence, the current research is mostly focused on a holistic approach of a simultaneous increase in production performance along with the disease resistance traits [4].

The increasing demand for eggs and poultry meat to meet the recommended nutritional requirement paves the way for rearing of alternate poultry species *viz*. ducks and quail which are known for their ability to produce more eggs and better meat as compared to chicken. The quail is an efficient egg and meat producer (unique flavor) having rapid growth, early sexual maturity, shorter generation interval, a higher rate of laying, early marketing age and low maintenance cost in comparison to chicken. The present concept of sustainable production requires optimum production performance along with giving appropriate weight age to disease resistance and health-related traits.

*Mx1* gene is an interferon-induced gene that inhibits the proliferation of avian influenza virus. However, very few reports are available on Japanese quail *Mx1* gene. Therefore, in the present study, we have tried to explore the genetic polymorphism of *Mx1* gene of Japanese quail using polymerase chain reaction-single-strand conformation polymorphism (PCR-SSCP) and nucleotide sequencing techniques.

## Materials and Methods

### Ethical approval

All the procedures have been conducted in accordance with the guidelines laid down by the Institutional Animal Ethical Committee of Indian Veterinary Research Institute.

### Resource population and sample collection

Total 170 adult Japanese quail birds maintained at Central Avian Research Institute (CARI), Izatnagar, and Bareilly were selected for sample collection. About 2 ml of blood sample was collected from each bird with EDTA as anticoagulant. The blood samples were kept in the deep freezer till DNA isolation.

### Amplification of exonic regions

The genomic DNA was isolated from the collected blood samples by conventional method [5]. The quality and purity of DNA was assessed by agarose gel electrophoresis and spectrophotometer, respectively. The genomic DNA was diluted to a concentration of 50 ng/µl. For PCR, the primers were designed [6,7] on the basis of available sequences of chicken (Acc No - DQ788613) and common quail (Acc No - EF575605) in public domain of NCBI for four different regions of *Mx1* gene. The PCR reactions were carried out in a total volume of 25 µl solution containing 1 µl of each forward and reverse primer (10 pmole/µl), 12.5 µl mastermix (MBI Fermentas), 1-2 µl genomic DNA (final concentration 60-90 ng/µl) and nuclease free water to make final volume. The annealing temperature for different fragments was optimized (Table-1). The amplification products were separated on 1.5% agarose gel electrophoresis, stained with 5 μg/ml of ethidium bromide with a 100 bp DNA ladder as molecular weight marker.

**Table-1:** Primer sequences and annealing temperature used to amplify *Mx1* gene in Japanese quail.

Fragments	Fragment size	Primer	Primer sequence (5’ → 3’)	Primer length (bp)	Annealing temperature (°C)
I (Exon 3)	185	Forward	GCAGCAGAACACAGCTTTCA	20	61
		Reverse	CTAGGAAGAGCAACACCAGAC	21	
II (Exon 5)	148	Forward	CAGGATATAGTGGCTAGCAC	20	56
		Reverse	GGTCATTATCTTGTGGCTGGTTCC	24	
III (Exon 7)	161	Forward	TCCTCACTAAACCAGATCTGGTG	23	59.2
		Reverse	TGCTGGATTACAGAGGCCAAGGA	24	
IV (Exon 13)	176	Forward	GCAAGCAACAGCTGCGAAAA	20	61.2
		Reverse	AAACCATTTCCAGGGCAAAGCTGG	24	

### Nucleotide polymorphism and DNA sequencing

The single nucleotide polymorphisms (SNPs) of *Mx1* gene were identified by PCR-SSCP technique [8,9]. The PCR products were resolved on 15% polyacrylamide gel. About 6 µl of PCR product and 12 µl of denaturing formamide dye (formamide, 95%; xylene cyanol, 0.025%; bromophenol blue, 0.025%; 0.5 M EDTA, 4%) were taken in a 0.2 ml PCR tube and mixed properly. The mixture of PCR product and formamide dye were denatured at 95°C for 10 min (by keeping in hot water bath) and snap chilled on ice for 15 min. The product was loaded in gel carefully. The electrophoresis was performed at 4°C for 13-16 h at 130 constant volts. For visualization of bands, silver staining was carried out. The pattern of DNA bands were documented by gel documentation system. The genotypes were identified, and the different SNPs were scored on banding pattern of SSCP. The gene and genotype frequencies were estimated [10]. The identified genotypes were custom sequenced and analyzed by BLAST (www.ncbi.nlm.nih.gov/BLAST). The nucleotide sequences and chromatograms were aligned and evaluated using BioEdit v7.0.5 [11]. The phylogenetic trees were constructed using MEGA 6 [12].

## Results and Discussion

### Polymorphism of *Mx1* gene

Out of the four fragments studied, Fragment II was found to be polymorphic by SSCP. Four different SSCP genotypes *viz*. AA, BB, CC, and DD were identified. The genotype frequency was found to be the highest for BB genotype (0.44) followed by AA (0.24), CC (0.18), and DD (0.14) genotype. The allele frequency was found to be in the decreasing order from B, A, C, and D (Table-2). However, the SSCP analysis could not reveal any polymorphism in three fragments (Fragment I, III, and IV).

**Table-2:** Allele-wise genotype and gene frequency in Fragment II.

Genotype	Genotype frequency	Allele	Gene/allele frequency
AA	0.24	A	0.24
BB	0.44	B	0.44
CC	0.18	C	0.18
DD	0.14	D	0.14

### Nucleotide sequence analysis

**Figure-1:**
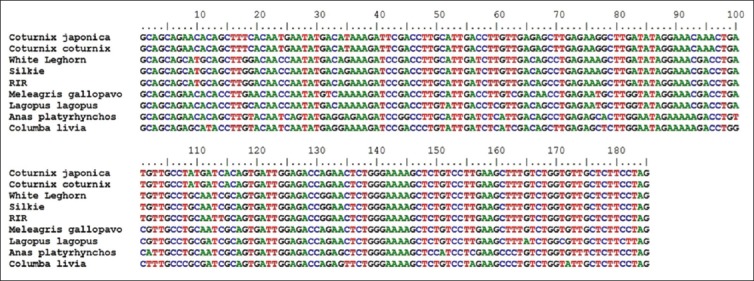
Aligned nucleotide sequence of 185 bp fragment of *Mx1* gene.

**Figure-2:**
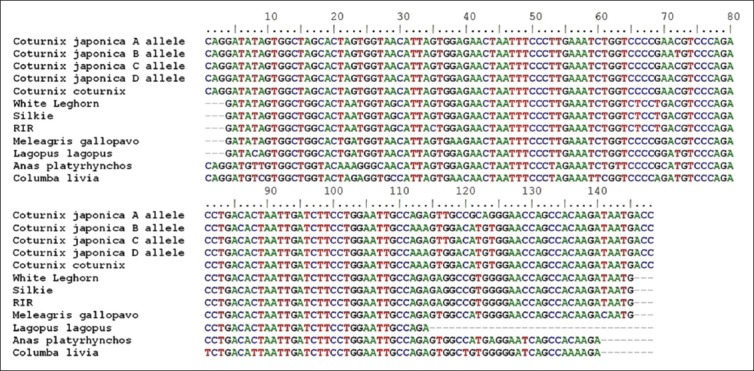
Aligned nucleotide sequence of 148 bp fragment of *Mx1* gene.

**Figure-3:**
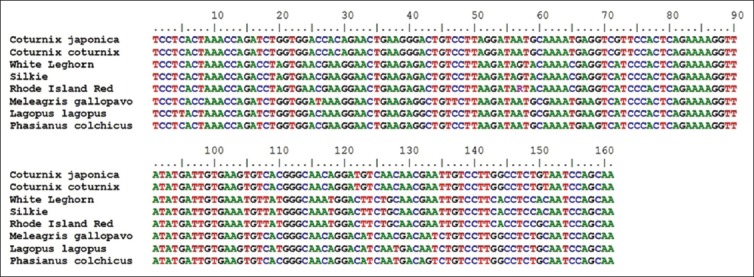
Aligned nucleotide sequence of 161 bp fragment of *Mx1* gene.

**Figure-4:**
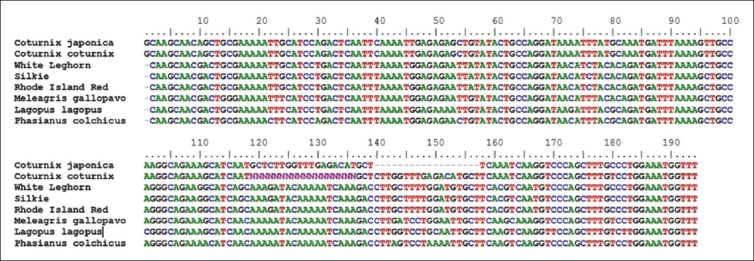
Aligned nucleotide sequence of 176 bp fragment of *Mx1* gene.

The amplified fragments of *Mx1* gene (Fragment I of 185 bp, Fragment II of 148 bp, Fragment III of 161 bp, and Fragment IV of 176 bp) of Japanese quail were custom sequenced [13] and were submitted to NCBI GenBank (KC571220, KC571221, KC571222, KC571223, KC571224, KC571225, and KC571226). All the sequences of Japanese quail as well as corresponding reported sequences of common quail, different breeds of chicken *viz*. RIR (NCBI Acc. No. DQ788613), SILKIE (NCBI Acc. No. DQ788614), WLH (NCBI Acc. No. DQ788615), *Phasianus colchicus* (Pheasant), *Meleagris gallopavo* (Turkey), *Columba livia* (Pigeon), and *Lagopus lagopus* (Willow ptarmigan) were aligned (Figure-1-4).

Between the Japanese quail and common quail, eight SNPs were identified in Fragment II, one SNP was identified in Fragment III, and no SNP was identified in fragment No. I and IV. However, between the quail (Japanese quail and common quail) and chicken breeds 94 SNPs were identified, out of which, 10 were species specific (Tables-3-6). In the 5^th^ exon (Fragment II) where four different alleles were identified) was found to be highly polymorphic and most of its nucleotide substitutions are non-synonymous. The allele “A” of 5^th^ exon of Fragment II in Japanese quail appeared to have maximum nucleotide substitutions as compared to other three alleles.

**Table-3:** Nucleotide substitutions in Fragment I (Exon 3 of Japanese quail *Mx1* gene).

Position (bp)	*Coturnix japonica*	*Coturnix coturnix*	WLH	Silki	RIR	Turkey	Willow ptarmigan	Duck	Pigeon
8	A	A	C	C	C	A	A	A	A
9	A	A	A	A	A	A	A	A	G
10	C	C	T	T	T	C	C	C	C
11	A	A	G	G	G	A	A	A	A
12	C	C	C	C	C	C	C	C	T
14	G	G	G	G	G	C	C	G	C
18	T	T	G	G	G	G	G	G	G
19	C	C	G	G	G	A	G	T	T
24	T	T	C	C	C	C	C	T	T
25	G	G	C	C	C	C	C	C	C
27	A	A	A	A	A	A	A	G	A
32	A	A	A	A	A	G	A	A	A
33	C	C	C	C	C	C	C	G	G
34	A	A	A	A	A	A	A	G	G
35	T	T	G	G	G	A	A	A	A
36	A	A	A	A	A	A	A	G	A
42	T	T	C	C	C	C	C	G	C
45	A	A	A	A	A	A	A	G	A
48	T	T	T	T	T	T	T	C	T
51	C	C	C	C	C	C	T	C	T
57	C	C	T	T	T	T	C	T	C
60	T	T	T	T	T	T	C	C	C
61	G	G	G	G	G	G	G	A	A
63	T	T	T	T	T	C	T	T	C
66	G	G	C	C	C	C	C	C	C
68	G	G	G	G	G	A	G	G	G
70	T	T	C	C	C	C	C	C	T
76	A	A	A	A	A	A	A	G	G
77	G	G	A	A	A	T	T	C	C
78	G	G	G	G	G	G	G	A	T
83	A	A	A	A	A	G	G	G	G
84	T	T	T	T	T	T	T	A	A
89	G	G	G	G	G	G	G	A	A
93	C	C	C	C	C	C	C	A	A
94	A	A	G	G	G	G	G	G	G
96	A	A	C	C	C	C	C	C	C
100	A	A	A	A	A	A	A	T	G
101	T	T	T	T	T	C	C	C	C
102	G	G	G	G	G	G	G	A	T
108	T	T	T	T	T	T	T	T	C
109	A	A	G	G	G	G	G	G	G
110	T	T	C	C	C	C	C	C	C
111	G	G	A	A	A	A	A	A	A
114	C	C	C	C	T	C	C	C	C
115	A	A	G	G	G	G	G	G	G
131	A	A	G	G	G	A	A	A	A
134	A	A	A	A	A	A	A	G	G
135	C	C	C	C	C	C	C	C	T
150	T	T	T	T	T	T	T	C	T
151	G	G	G	G	G	G	G	A	G
156	T	T	T	T	T	T	T	C	A
162	T	T	T	T	T	T	T	C	C
163	T	T	T	T	T	T	T	C	C
165	G	G	G	G	G	G	A	G	G
171	T	T	T	T	T	T	C	T	T
172	G	G	G	G	G	G	G	G	A
175	G	G	G	G	G	G	G	T	G
182	C	C	C	C	C	C	T	C	C

**Table-4:** Nucleotide substitutions in Fragment II (Partial exon 5 of Japanese quail *Mx1* gene).

Position (bp)	*Coturnix japonica*				*Coturnix coturnix*	WLH	Silki	RIR	Turkey	Willow ptarmigan	Duck	Pigeon

	A Allele	B Allele	C Allele	D Allele								
7	A	A	A	A	A	A	A	A	A	A	G	G
8	T	T	T	T	T	T	T	T	T	C	T	T
9	A	A	A	A	A	A	A	A	A	A	T	T
16	A	A	A	A	G	G	G	G	G	G	G	G
18	C	C	C	C	C	C	C	C	C	C	T	T
21	T	T	T	T	T	T	T	T	T	T	A	T
22	A	A	A	A	A	A	A	A	G	G	A	A
23	G	G	G	G	A	A	A	A	G	A	A	G
24	T	T	T	T	T	T	T	T	T	T	G	A
27	T	T	T	T	T	T	T	T	T	T	C	T
28	A	A	A	A	A	A	A	A	A	A	A	G
29	A	A	A	A	A	G	G	G	A	A	A	C
35	G	G	G	G	G	G	G	C	G	G	G	G
38	G	G	G	G	G	G	G	G	A	G	G	A
40	G	G	G	G	G	G	G	G	G	G	G	C
54	T	T	T	T	T	T	T	T	T	T	A	A
60	C	C	C	C	C	C	C	C	C	C	C	T
61	T	T	T	T	T	T	T	T	T	T	T	C
63	G	G	G	G	G	G	G	G	G	G	T	G
66	C	C	C	C	C	T	T	T	C	C	C	C
69	G	G	G	G	G	T	T	T	G	G	G	G
70	A	A	A	A	A	G	G	G	G	G	C	G
72	C	T	C	C	C	C	C	C	C	T	T	T
81	C	C	C	C	C	C	C	C	C	C	C	T
88	C	C	C	C	C	C	C	C	C	C	C	T
113	G	A	G	A	A	G	G	G	G	G	G	G
116	T	T	T	T	T	A	A	A	T	-	T	T
117	T	G	T	G	G	G	G	G	G	-	G	G
119	C	A	A	A	A	C	C	C	C	-	C	C
121	G	A	A	A	A	G	G	G	A	-	A	G
122	C	T	T	T	T	T	T	T	T	-	T	T
123	A	G	G	G	G	G	G	G	G	-	G	G
124	G	T	T	T	T	G	G	G	G	-	A	G
127	A	A	A	A	A	A	A	A	A	-	G	G
129	C	C	C	C	C	C	C	C	C	-	T	T
136	C	C	C	C	C	C	C	C	C	-	C	A
141	T	T	T	T	T	T	T	T	C	-	-	-

**Table-5:** Nucleotide substitutions in Fragment III (partial exon 7 of Japanese quail *Mx1* gene).

Position	*Coturnix japonica*	*Coturnix coturnix*	WLH	Silki	RIR	Turkey	Willow ptarmigan	Pheasant
5	C	C	C	C	C	C	T	C
8	T	T	T	T	T	C	T	T
17	T	T	C	C	C	T	T	T
20	G	G	A	A	A	G	G	G
24	G	G	A	A	A	G	G	G
26	C	C	C	C	C	T	C	C
27	C	C	G	G	G	A	A	G
29	C	C	A	A	A	A	A	A
30	A	A	G	G	G	G	G	G
40	G	G	A	A	A	A	A	A
42	A	A	A	A	A	G	G	G
47	C	C	C	C	C	T	C	C
52	G	G	A	A	A	A	A	A
57	A	A	G	G	G	A	A	A
59	G	G	A	A	A	G	G	G
61	A	A	A	A	A	G	G	A
65	T	T	C	C	C	T	T	T
68	G	G	G	G	G	A	A	A
72	G	G	A	A	A	A	A	A
74	T	T	C	C	C	C	C	C
104	G	G	A	A	A	G	G	G
108	C	C	T	T	T	C	C	C
110	C	C	T	T	T	C	T	C
117	C	C	A	A	A	C	C	C
118	A	A	T	T	T	A	A	A
122	T	T	C	C	C	C	C	C
123	G	G	T	T	T	A	A	A
126	A	A	T	T	T	A	A	A
127	A	A	G	G	G	A	A	A
128	C	C	C	C	C	C	T	T
129	A	A	A	A	A	G	G	G
132	G	G	G	G	G	A	A	A
133	A	A	A	A	A	A	A	G
134	A	A	A	A	A	T	T	T
135	T	T	T	T	T	C	C	C
143	G	G	C	C	C	G	G	G
144	G	G	A	A	A	G	G	G
149	G	G	C	C	C	G	G	G
150	G	G	A	A	G	G	G	G
151	T	T	C	C	C	C	C	C

**Table-6:** Nucleotide substitutions in Fragment IV (partial exon 13 of Japanese quail *Mx1* gene).

Position	*Coturnix japonica*	*Coturnix coturnix*	WLH	Silki	RIR	Turkey	Willow ptarmigan	Pheasant
10	A	A	G	G	G	G	G	G
11	G	G	A	A	A	A	A	A
15	C	C	C	C	C	G	C	C
22	T	T	T	T	T	T	T	C
24	G	G	G	G	G	T	T	T
30	A	A	T	T	T	T	T	T
40	C	C	T	T	T	T	T	T
46	T	T	G	G	G	G	G	G
51	G	G	G	G	G	G	A	G
53	G	G	A	A	A	A	A	A
54	C	C	T	T	T	T	T	C
56	G	G	A	A	A	G	G	G
73	A	A	C	C	C	C	G	C
76	T	T	C	C	C	T	T	T
79	T	T	C	C	C	C	C	C
80	G	G	A	A	A	A	G	G
83	A	A	G	G	G	G	G	G
96	T	T	C	C	C	C	C	C
101	A	A	A	A	A	A	C	A
102	A	A	G	G	G	G	G	G
110	A	A	G	G	G	A	A	A
111	G	G	G	G	G	G	G	A
117	A	A	G	G	G	A	A	A
118	T	T	C	C	C	C	C	C
161	A	A	C	C	C	A	A	A
162	A	A	G	G	G	G	G	G
163	T	T	T	T	T	C	T	T
164	C	C	C	C	C	A	C	C
167	G	G	T	T	T	G	G	G
170	C	C	C	C	C	C	T	C
180	C	T	C	C	C	T	T	T
182	C	C	C	C	C	C	T	C

**Figure-5:**
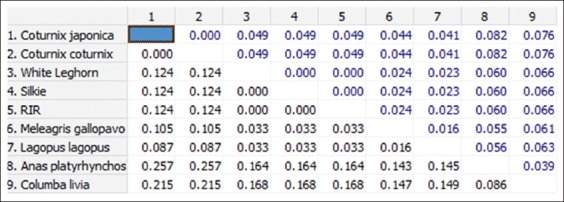
Nucleotide sequence distance of 185 bp fragment of *Mx1* gene between different species (The number of base substitutions per site between sequences is shown. Standard error estimate(s) are shown above the diagonal and were obtained by a bootstrap procedure 1000 replicates. Analyses were conducted using the Kimura 2-parameter model).

**Figure-6:**
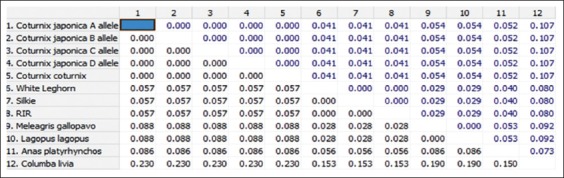
Nucleotide sequence distance of 148 bp fragment of *Mx1* gene between different species (The number of base substitutions per site between sequences is shown. Standard error estimate(s) are shown above the diagonal and were obtained by a bootstrap procedure 1000 replicates. Analyses were conducted using the Kimura 2-parameter model).

**Figure-7:**
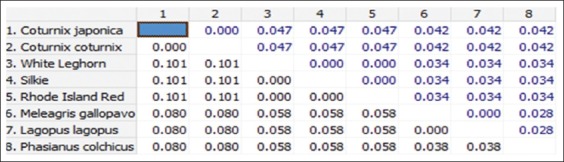
Nucleotide sequence distance of 161 bp fragment of *Mx1* gene between different species (The number of base substitutions per site between sequences is shown. Standard error estimate(s) are shown above the diagonal and were obtained by a bootstrap procedure, 1000 replicates. Analyses were conducted using the Kimura 2-parameter model).

**Figure-8:**
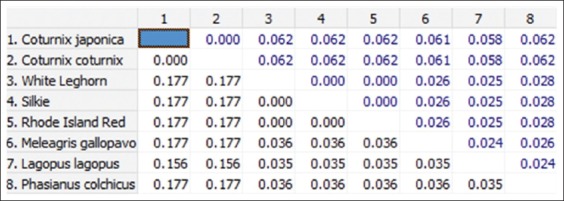
Nucleotide sequence distance of 176 bp fragment of *Mx1* gene between different species (The number of base substitutions a site between sequences is shown. Standard error estimate(s) are shown above the diagonal and were obtained by a bootstrap procedure, 1000 replicates. Analyses were conducted using the Kimura 2-parameter model).

Sequence divergence analysis using MEGA 6 with 1000 replicates of bootstrap and Kimura 2 parameter model revealed that sequence of Japanese quail is almost 100% identical to that of common quail (Figures-5-8) in all the four fragments. However, the sequence of Japanese quail showed divergence of 12.4%, 5.7%, 10.1%, and 17.7% from sequence of chicken as well as 10.5%, 8.8%, 8.0%, and 17.7% from sequence of turkey in Fragment I, II, III, and IV, respectively (Figures-5-8).

**Figure-9:**
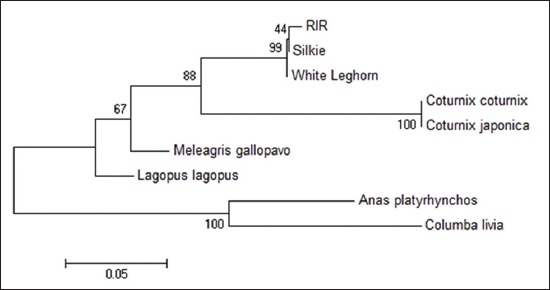
Phylogenetic tree based on 185 bp fragment nucleotide sequence of *Mx1* gene.

**Figure-10:**
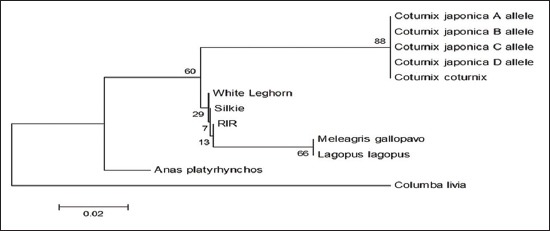
Phylogenetic tree based on 148 bp fragment nucleotide sequence of *Mx1* gene.

**Figure-11:**
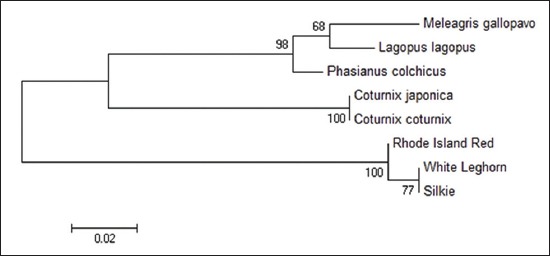
Phylogenetic tree based on 161 bp fragment nucleotide sequence of *Mx1* gene.

**Figure-12:**
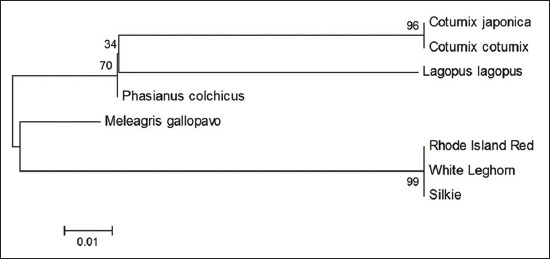
Phylogenetic tree based on 176 bp fragment nucleotide sequence of *Mx1* gene.

The phylogenetic tree analysis of the amplified sequence of four fragments revealed that Japanese quail and common quail always remains in the same cluster indicating their common ancestral origin (Figures-9-12). The deduced amino acid sequences from the nucleotide sequences of four fragments from Japanese quail, common quail, and chicken were analyzed for sequence homology. Within quail sequences, only four amino acid substitutions were observed (present in Fragment II only). However, between quail and chicken, 56 (12+10+14+20) amino acid substitutions were identified. Clustering of chicken sequences in one cluster along with grouping of common quail and Japanese quail in other cluster is very well expected as per taxonomic classification keeping Japanese quail (*Coturnix Japonica*) and common quail (*Coturnix coturnix*) in the one genus *Coturnix*, whereas chicken in another genus *Gallus* of one family Phasianidae [14].

## Conclusion

The *Mx1* gene was found to be polymorphic in Japanese quail in one of the four fragments studied. The B allele was predominant, out of the three alleles found in 148 bp fragment of *Mx1* gene. Analysis of four different fragments showed that Japanese quail *Mx1* gene showed relatively high degree of homology with other poultry species. The relative conserve nature of *Mx1* gene across the species confirms its biological role as an immunity-related gene. The other regions of this gene need to be sequenced and association with disease resistance traits may be done for the complete characterization of this gene in Japanese quail.

## Authors’ Contributions

PK, BB, TKB, and AKT planned and designed the experiment. DK conducted the experimental work. RN and VKS collected the blood samples. CM, NRS, KS, and DS were involved in scientific discussion and analysis of the data. All authors read and approved the final manuscript.
